# Assistance by Routine CT Features Combined With 3D Texture Analysis in the Diagnosis of BRCA Gene Mutation Status in Advanced Epithelial Ovarian Cancer

**DOI:** 10.3389/fonc.2021.696780

**Published:** 2021-07-26

**Authors:** Meng-ru Li, Ming-zhu Liu, Ya-qiong Ge, Ying Zhou, Wei Wei

**Affiliations:** ^1^ Department of Radiology, Anhui Provincial Hospital Affiliated to Anhui Medical University, Hefei, China; ^2^ Department of Radiology, The First Affiliated Hospital of University of Science and Technology of China (USTC), Division of Life Sciences and Medicine, University of Science and Technology of China, Hefei, China; ^3^ General Electric (GE) Healthcare China, Shanghai, China; ^4^ Department of Gynecological Oncology, The First Affiliated Hospital of USTC, Hefei, China

**Keywords:** mutation status, epithelial ovarian cancer, texture analysis, routine CT feature, BRCA gene

## Abstract

**Purpose:**

To evaluate the predictive value of routine CT features combined with 3D texture analysis for prediction of BRCA gene mutation status in advanced epithelial ovarian cancer.

**Method:**

Retrospective analysis was performed on patients with masses occupying the pelvic space confirmed by pathology and complete preoperative images in our hospital, including 37 and 58 cases with mutant type and wild type BRCA, respectively (total: 95 cases). The enrolled patients’ routine CT features were analyzed by two radiologists. Then, ROIs were jointly determined through negotiation, and the ITK-SNAP software package was used for 3D outlining of the third-stage images of the primary tumor lesions and obtaining texture features. For routine CT features and texture features, Mann-Whitney U tests, single-factor logistic regression analysis, minimum redundancy, and maximum correlation were used for feature screening, and the performance of individual features was evaluated by ROC curves. Multivariate logistic regression analysis was used to further screen features, find independent predictors, and establish the prediction model. The established model’s diagnostic efficiency was evaluated by ROC curve analysis, and the histogram was obtained to conduct visual analysis of the prediction model.

**Results:**

Among the routine CT features, the type of peritoneal metastasis, mesenteric involvement, and supradiaphragmatic lymph node enlargement were correlated with BRCA gene mutation (P < 0.05), whereas the location of the peritoneal metastasis (in the gastrohepatic ligament) was not significantly correlated with BRCA gene mutation (P > 0.05). Multivariate logistic regression analysis retained six features, including one routine CT feature and five texture features. Among them, the type of peritoneal metastasis was used as an independent predictor (P < 0.05), which had the highest diagnostic efficiency. Its AUC, accuracy, specificity, and sensitivity were 0.74, 0.79, 0.90, and 0.62, respectively. The prediction model based on the combination of routine CT features and texture features had an AUC of 0.86 (95% CI: 0.79–0.94) and accuracy, specificity, and sensitivity of 0.80, 0.76, and 0.81, respectively, indicating a better performance than that of any single feature.

**Conclusions:**

Both routine CT features and texture features had value for predicting the mutation state of the BRCA gene, but their predictive efficiency was low. When the two types of features were combined to establish a predictive model, the model’s predictive efficiency was significantly higher than that of independent features.

## Introduction

Ovarian cancer is one the most lethal gynecological tumors, with epithelial ovarian cancer as the most common pathological type (1, 2). Studies have shown that about 50% of epithelial ovarian cancers have homologous recombination repair defects, and BRCA genes can participate in the repair of DNA double strand breaks through homologous recombination integration (3, 4). In addition, patients with BRCA gene mutations have a higher objective response rate and survival rate with platinum chemotherapies, and the BRCA gene mutation status is associated with survival and recurrence (5–7). Currently, genetic testing is the main way to identify BRCA mutations, but it is usually done on an individual basis and does not capture the full picture of the tumor (8). H. A. Vargas et al. (9) concluded that the state of the BRCA gene can be predicted by the routine CT features, but the results mainly depend on the observer’s subjective experience, and the accuracy was low. Recently, texture analysis (10, 11) has become a research hotspot in medical domain, By combining artificial intelligence with medical images, it can quantify the pixel and spatial distributions of medical images, capture the inherent heterogeneity of tumors, and correlate them with the underlying gene expression types.

Therefore, in this study, texture analysis was combined with routine CT features to establish a prediction model for detecting BRCA mutation status. The objective was to assist with the diagnosis of BRCA gene mutation types before surgery and guide the selection of chemotherapy drugs after surgery.

## Material and Methods

### Study Population

**Figure 1:**
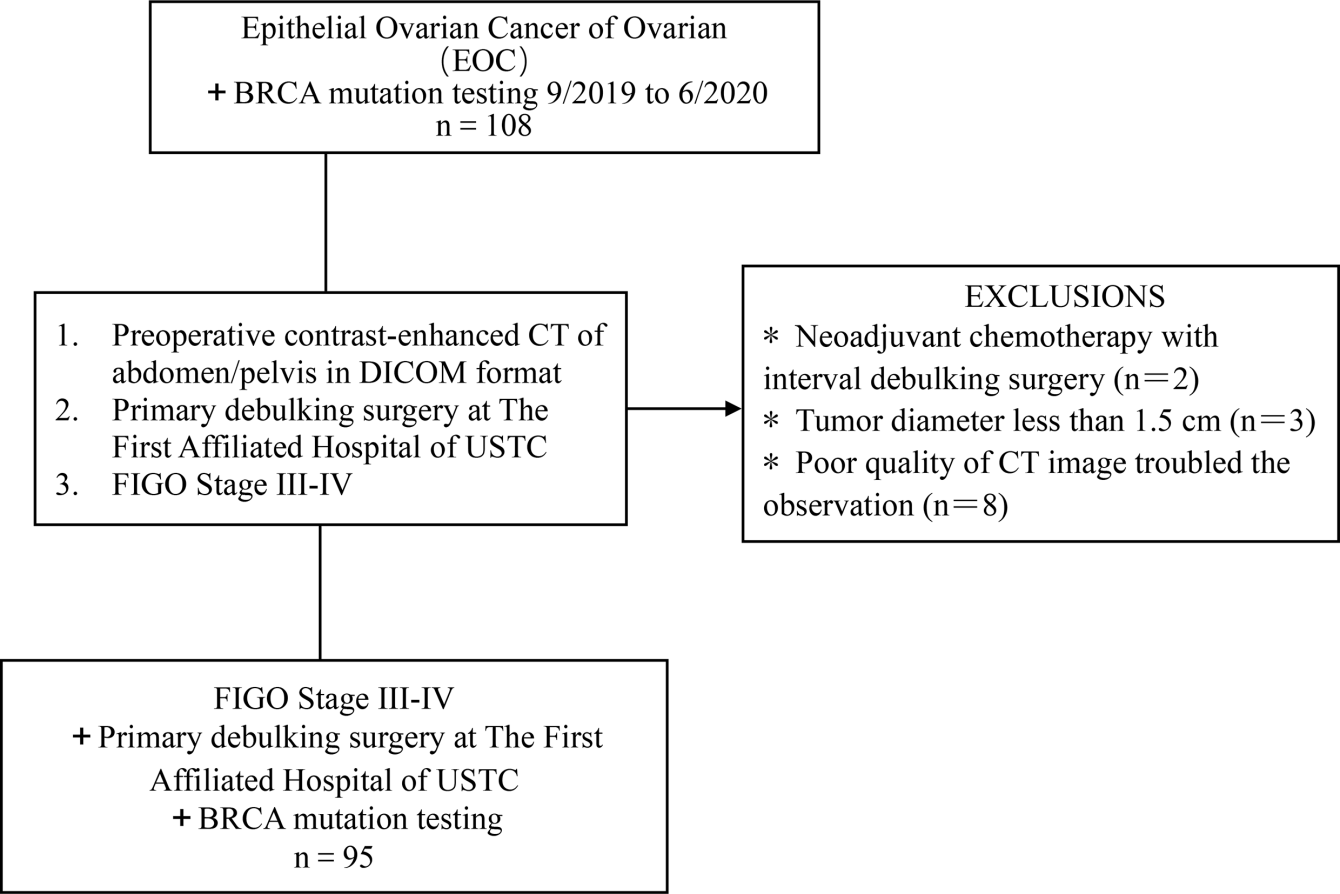
Flowchart shows the details of patient selection process. DICOM, Digital Imaging and Communications in Medicine, FIGO, International Federation of Gynecology and Obstetrics; USTC, University of Science and Technology of China.

Our Institutional Review Board waived written informed consent for this retrospective study, as only de-identified data were evaluated, and the study involved no potential risk to patients. The pathologic and imaging data of 108 cases of patients with ovarian cancer who were admitted to our hospital from September 2019 to June 2020 were retrospectively analyzed (including 63, 13, 15, and 4 cases of serous, mucinous, clear cell, and endometrioid carcinoma, respectively; 32 and 63 patients with stage III and IV cancer, respectively). Inclusion criteria: (1) advanced epithelial ovarian cancer was confirmed by surgery and histology; (2) NGS gene test was performed, and the test results were clear; (3) routine abdominal and pelvic enhanced CT examination was performed preoperatively or during chemoradiotherapy. In total, 13 patients were excluded owing to the following criteria: (1) tumor diameter less than 1.5 cm (n = 3); (2) The poor quality of CT image troubled the observation of radiologists (n = 8); (3) the patient underwent neoadjuvant chemotherapy before surgery (n = 2). Ninety-Five patients met the eligibility criteria (**Table 1** and **Figure 1**).

**Table 1:** Patients and Clinical Characteristics.

Characteristics	All (n = 95)	BRCA Mutant (n = 37)	BRCA Wild Type (n = 58)
Age at diagnosis (y)*	57 (36-79)	55 (36-75)	59 (38-79)
FIGO stage			
III	32 (34)	9 (24)	23 (40)
IV	63 (66)	28 (76)	35 (60)
Pathological Classification			
Serous	63 (66)	30 (81)	33 (57)
Mucinous	13 (14)	6 (16)	7 (12)
Clear cell carcinoma	15 (16)	1 (3)	14 (24)
Endometrial	4 (4)	0 (0)	4 (7)

FIGO, International Federation of Gynecology and Obstetrics.

*Data in parentheses are the range.

All patients received NGS gene testing when at least one of the following indications were present: (1) family history of pathogenic BRCA gene mutations; (2) family history of breast cancer before age 45 years and triple-negative breast cancer before age 60 years; (3) the patient’s economic condition permitted or the doctor required genetic testing on the basis of the patient’s condition. To ensure the consistency of test results, genetic counseling and gene sequencing were performed by the same testing institution for all subjects. Finally, the subjects were divided into mutant type and wild type BRCA status on the basis of the results of the NGS gene test. In addition, germline mutation was assessed in this study.

### Inspection Method

After fasting for more than 8h before imaging, the patients received oral water of about 500–1000 mL 15–30 min before examination. After the bladder was emptied, abdominal and pelvic plain scanning were performed on a GE Discovery CT 750HD (HDCT, USA) scanner. The patients were placed in supine position, and the scanning range was set from the diaphragmatic apex or iliac spine to the pubic joint. The tube voltage was 120 kV, the tube current was 280–300 mA, the rotation time was 0.5s, the detector combination was 0.625 × 64mm, the bed entry speed was 47.5 mm/s, and the layer thickness was 1.25 mm. Iodohexol (300 mgI/mL) was used as the contrast agent for enhanced scanning. The injection dose was 1.5 mL/kg body mass, and the flow rate was 3 mL/s. The contrast agent was injected at 25–30 s, 60–70 s, and 180–200 s before arterial, venous, and delayed scanning, respectively. The original CT images were obtained through the scanner’s Mxview Workstation in.DICOM (Digital Imaging and Communications in Medicine) format.

### Routine CT Features

**Figure 2:**
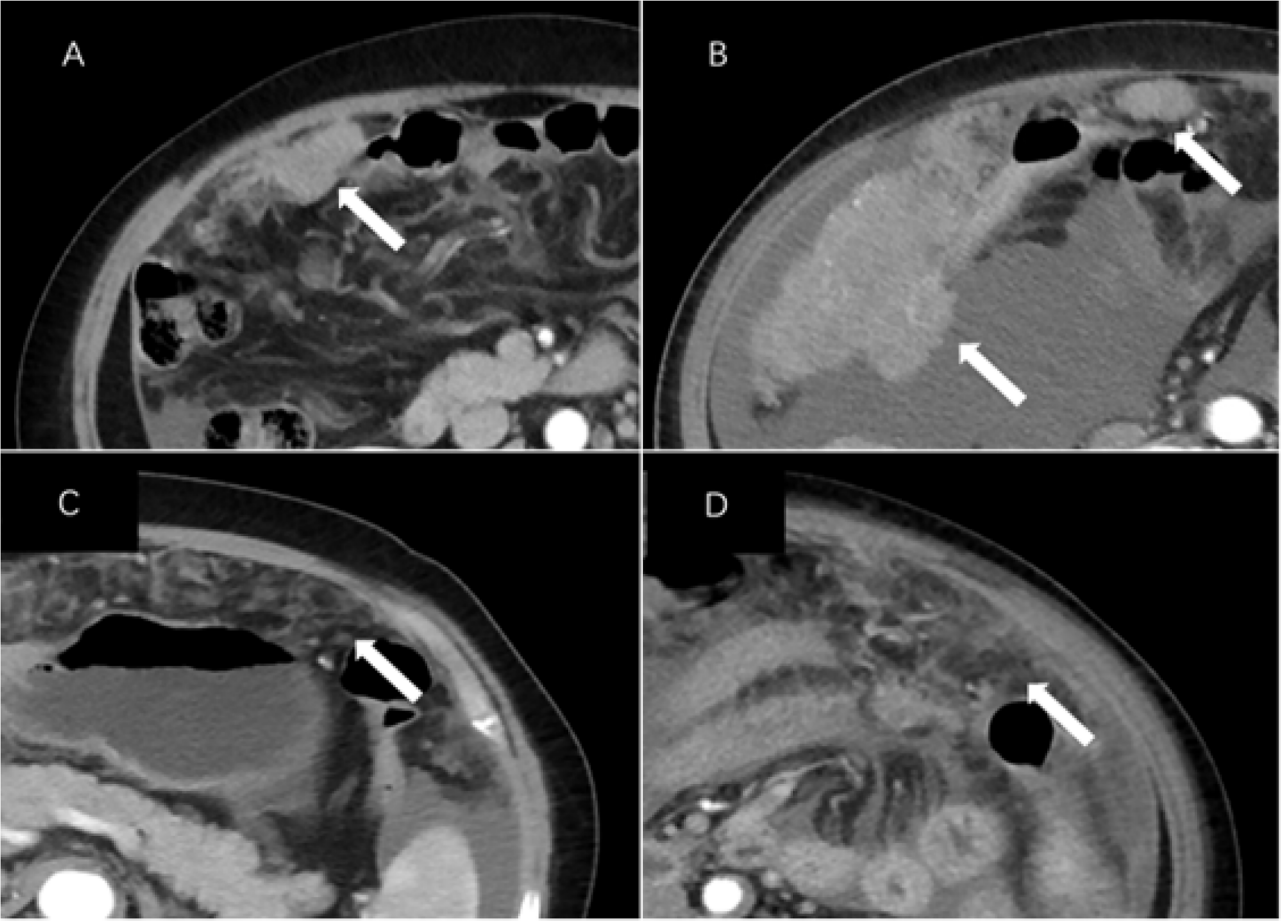
CT images of peritoneal metastases in a patient with epithelial ovarian cancer. Axial CT images of **(A)** a 66-year-old woman and **(B)** a 76-year-old woman with BRCA mutations (stage III and IV, respectively) showing nodular lesions (arrows). Axial CT images of **(C)** a 66-year-old woman and **(D)** a 68-year-old woman with invasive cancer and wild-type BRCA (both stage III, arrows).

The subjects’ preoperative enhanced CT images were analyzed retrospectively by two radiologists in our hospital. The results were recorded in an Excel spreadsheet. The primary tumor was assessed including lesion margins (smooth or irregular), lateral type (left/right/bilateral), architecture (cystic/predominantly cystic or solid/predominantly solid), and the presence or absence of calcification. Evaluation of lesions outside the ovary included ascites (none/small/large) (12), the type of peritoneal metastasis, mesenteric involvement, and enlarged lymph nodes. Mesenteric involvement manifests as mesenteric infiltration and/or nodules. Peritoneal metastases were categorized as nodular or infiltrative: a nodular metastasis was a well-defined soft tissue mass, whereas an invasive one was not well defined or flocculent (**Figure 2**). Distant sites of metastasis included the liver, spleen, and lesser omentum sac (hepatogastric ligament) (13). The location of enlarged lymph nodes was also assessed: (a) supradiaphragmatic, or (b) perihepatic. Each node was evaluated by the observer, and enlarged lymph nodes were defined by the shortest diameter of the lymph node (14): (a) supradiaphragmatic lymph nodes, >0.5 cm; (b) lymph nodes located around the portal vein (hilar part of the liver), >1.0 cm.

### ROI Segmentation and Extraction of Texture Features

**Figure 3:**
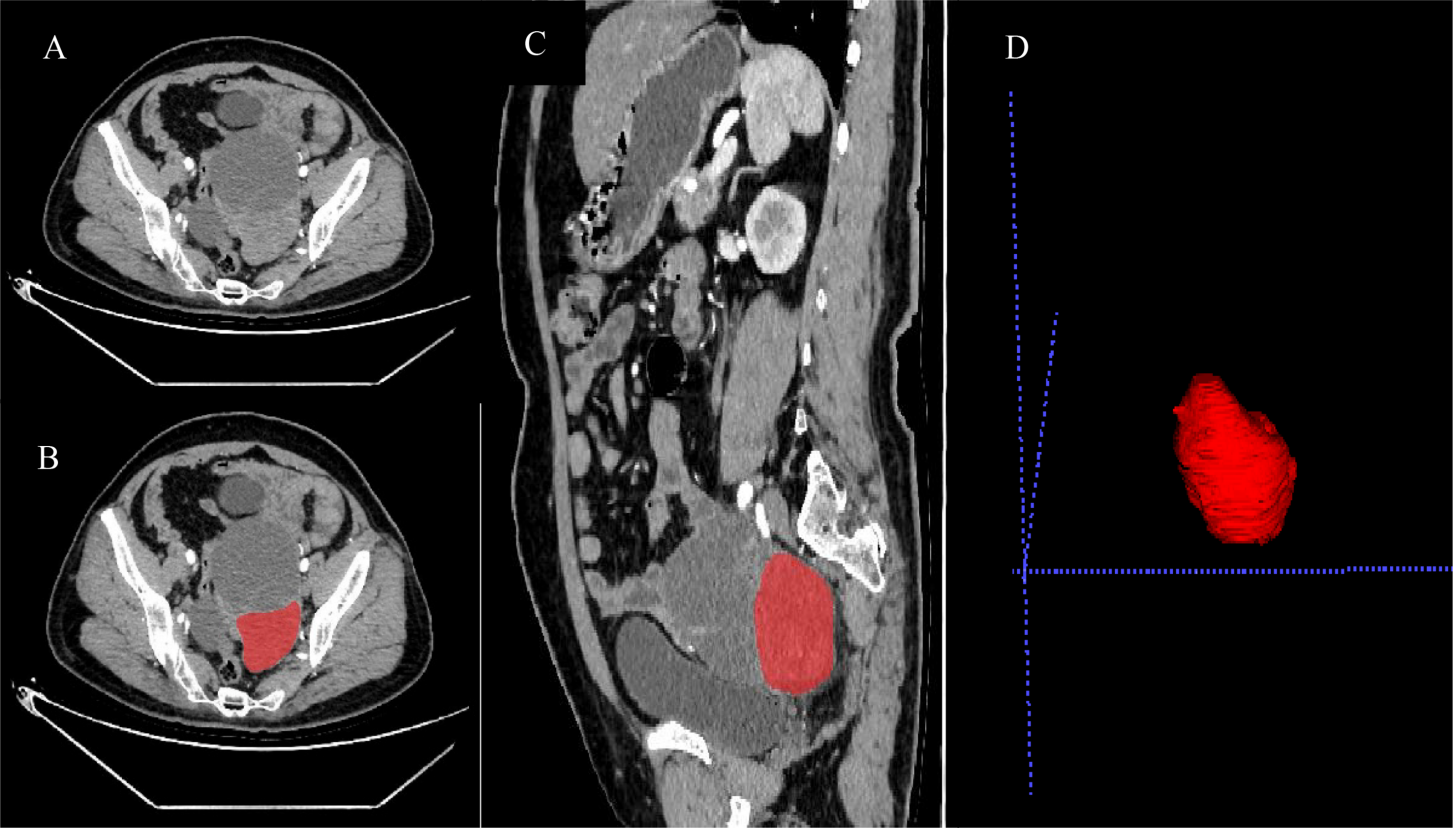
Preoperative enhanced CT arterial phase images of patients with wild-type BRCA **(A)**, as well as processing images **(B–D)** after ROIs were drawn by ITK-SNAP software.

The locations of the 3D tumor lesions were determined by consultation between two radiologists in our hospital. ROIs of all patients’ arterial phase, venous phase, and delay phase images were outlined in the ITK-SNAP software package(https://www.itksnap.org) (**Figure 3**). Before drawing the ROIs, the grayscale range was adjusted to a unified range (window width, 300 HU; window position, 30HU) including all primary tumor lesions visible to the naked eye as far as possible. Attention was paid to avoiding vascular shadows, calcification, and other areas during drawing. The original image and ROI image guide were imported into A. K. software (GE medical, Shanghai, China), which can automatically identify matching between two sets of images and conduct pattern feature extraction. Finally, the software extracted 653 features and provided 6 major types of analysis: histograms, a gray unicom regional matrix, neighborhood grayscale difference matrix, gray level co-occurrence matrix, morphology, and a run-length matrix.

### Statistical Analysis

All data were counted and processed by the R 3.5.1 software package (http://www.Rproject.org). (1) First, the extracted texture features and routine CT features were tested by Mann-Whitney U test and single-factor logistic regression analysis, and the texture features that could distinguish the patients with mutant and wild-type BRCA were screened (P < 0.05). Then, using minimum redundancy and maximum correlation analysis, the redundant features were eliminated, the features that had strong correlations with the two groups of tags were retained, and the discrimination performance of the 20 features with good performance was analyzed. (2) Multiple logistic regression analysis was then adopted to identify features that were independent predictors and establish a prediction model, and the model’s performance was evaluated with ROC curve analysis. (3) The multiple logistic regression model was visualized using a line chart. (4) The data were randomly divided into training and validation sets in a 7:3 ratio, and 100 iterations of resampling using the cross-validation method were applied to verify the integrated. model’s stability and reliability. Then, the model’s accuracy, sensitivity, and specificity on the training set and the validation set were calculated.

## Results

The Mann-Whitney U test results showed that mesenteric involvement, the type of peritoneal metastasis, and supradiaphragmatic lymph node enlargement were statistically significant (P < 0.05) (**Table 2**). In univariate logistic regression analysis, the imaging features that differentiated the wild type from the mutant BRCA gene included mesenteric involvement (OR = 1.643), peritoneal metastasis (OR = 2.800), and supradiaphragmatic enlarged lymph nodes (OR = 1.800) (**Table 3**).

**Table 2:** Mann-Whitney U test results of correlation between imaging features and BRCA mutation status.

Imaging features	BRCA		
	BRCA wild type	BRCA mutation type	P value
Side			0.624
Left	14 (24.14%)	12 (32.43%)	
Right	20 (34.48%)	9 (24.32%)	
Both	24 (41.38%)	16 (43.25%)	
Margins			0.089
Smooth	19 (32.76%)	5 (13.51%)	
Irregular	39 (67.24%)	32 (86.49%)	
Architecture			0.673
Cystic/predominantly cystic	22 (37.93%)	15 (40.54%)	
Solid/predominantly solid	36 (62.07%)	22 (59.46%)	
Calcification			0.568
Present	8 (13.79%)	6 (16.22%)	
Absent (reference)	50 (86.21%)	31 (83.78%)	
Ascites			0.167
None	13 (22.41%)	4 (10.81%)	
Small	25 (43.10%)	15 (40.54%)	
Large	20 (34.49%)	17 (48.65%)	
Distant metastases			
Liver metastasis			0.177
Present	12 (20.69%)	13 (35.14%)	
Absent (reference)	46 (79.31%)	24 (64.86%)	
Spleen metastasis			0.6673
Present	9 (15.52%)	7 (18.92%)	
Absent (reference)	49 (84.48%)	30 (81.08%)	
Gastrohepatic ligament			0.753
Present	14 (24.14%)	10 (27.03%)	
Absent (reference)	44 (75.86%)	27 (72.97%)	
Peritoneum			
peritoneal metastasis	52 (89.66%)	29 (78.38%)	< 0.01
Nodular	14 (26.92%)	23 (79.31%)	
Infiltrative	38 (73.08%)	6 (20.69%)	
Absent (reference)	6 (10.34%)	8 (21.62%)	
Mesentery			0.036
Present	10 (17.24%)	12 (32.43%)	
Absent (reference)	48 (82.76%)	25 (67.57%)	
Lymphadenopathy			
Supradiaphragmatic			0.006
Present	10 (17.24%)	16 (43.24%)	
Absent (reference)	48 (82.76%)	21 (56.76%)	
Periportal			0.099
Present	9 (15.52%)	9 (24.32%)	
Absent (reference)	49 (84.48%)	28 (75.68%)	

No ascites, defined as the surgical record clearly indicating no ascites; A small amount of ascites, defined as <1500 mL; Massive ascites, defined as ≥1500 mL (12).

**Table 3:** Single-factor logistic regression analysis of correlation between CT features and BRCA mutation status.

Imaging features	OR value	P value
Mesenteric involvement	1.64336	0.04095
Form of peritoneal metastasis	2.79907	0.00005
Margins	1.42469	0.09119
Enlarged lymph nodes above the diaphragm	1.78817	0.00697

**Figure 4:**
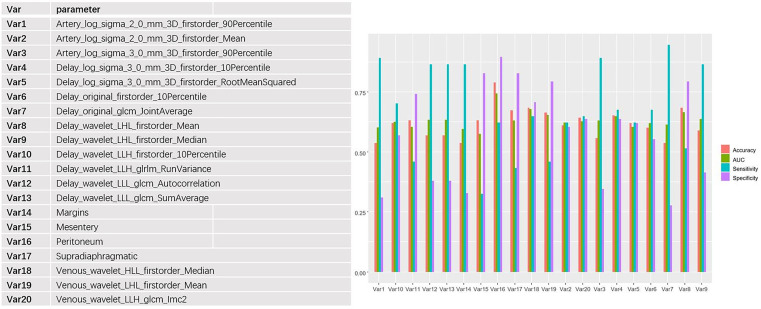
Independent predictive efficacy of the 20 features with the best performance (including CT imaging features and texture features), among which the peritoneal transition form had the best diagnostic efficacy, with AUC, accuracy, specificity, and sensitivity of 0.74, 0.79, 0.90, and 0.62, respectively.

The 653 extracted texture features were also tested by Mann-Whitney U tests and single-factor logistic regression analysis. Then, the conventional CT image features and texture features were selected by the minimum redundancy and maximum correlation method. The features’ specific independent diagnostic efficiency is shown in **Figure 4**. The peritoneal metastasis form had the best diagnostic efficiency, with an AUC value of only 0.74, but it had a poor sensitivity (0.62).

**Figure 5:**
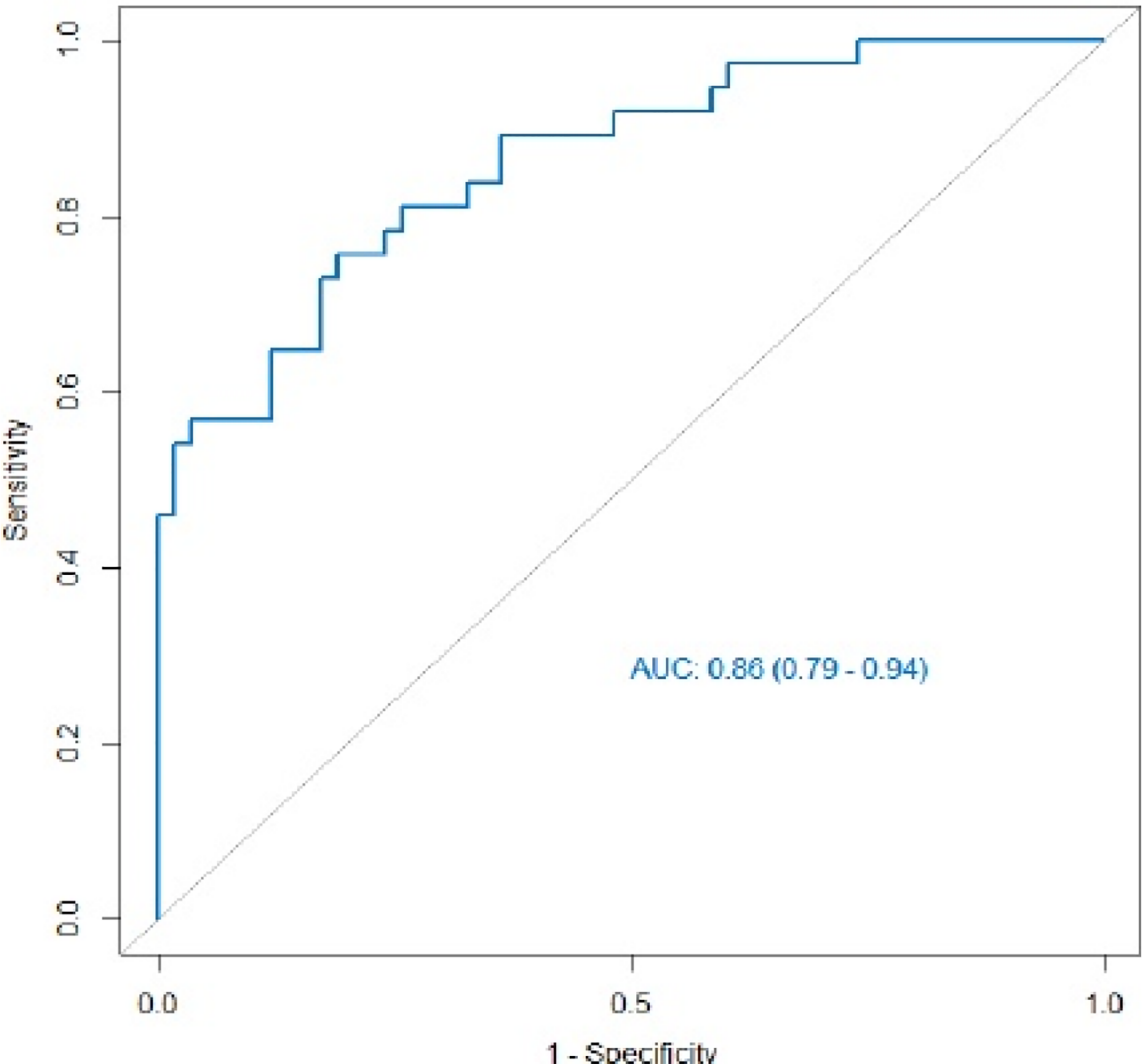
ROC curve of the integrated model. Its AUC value was 0.86 (95% CI 0.79–0.94), and its prediction performance was significantly improved compared with that of the single feature.

**Figure 6:**
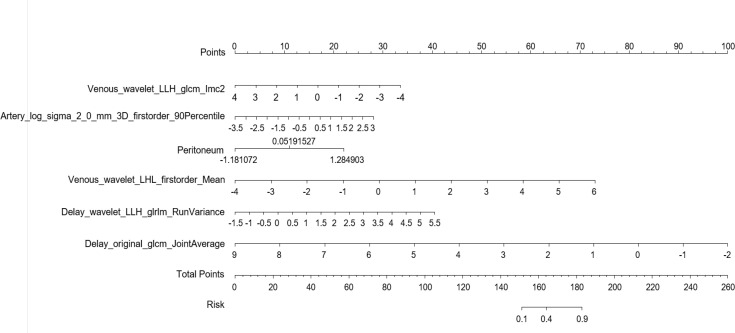
Establishment of a nomogram risk model to predict BRCA gene mutation status in epithelial ovarian cancer.

Multiple logistic regression analysis was conducted to screen independent discriminant features and build a prediction model. Eventually incorporated into the model were features including identities, odds ratios (OR values), and P values (**Table 4**). Patients with OR values of >1 had a greater probability of mutation, and this was an independent predictor of the characteristics of ovarian cancer (P < 0.05), in which the type of peritoneal metastasis was an independent predictor. The type of peritoneal metastasis was also an independent predictor of BRCA mutation state, and the integration model of BRCA mutation state in ovarian cancer had better prediction performance (**Figure 5** and **Table 5**). In the resulting nomogram model, the feature values of texture features in the optimal feature subset were significantly higher than those of conventional CT image features (**Figure 6**).

**Table 4:** Multifactorial analysis of correlations: CT features and texture features vs. BRCA mutation status.

Optimal feature subset	5%	95%	OR value	P value
Venous_wavelet_LLH_glcm_Imc2	0.25	1.24	0.56	0.1545
Artery_log_sigma_2_0_mm_3D_ firstorder_90Percentile	0.87	3.76	1.81	0.1103
Form of peritoneal metastasis	1.82	6.44	3.42	0.0001
Venous_wavelet_LHL_firstorder_Mean	0.63	11.88	2.74	0.1782
Delay_wavelet_LLH_glrlm_RunVariance	0.99	4.96	2.22	0.0521
Delay_original_glcm_JointAverage	0.05	1.63	0.29	0.1577

**Table 5:** Diagnostic effectiveness of the integrated model.

	Threshold	Accuracy	Sensitivity	Specificity	Positive Predictive Value	Negative Predictive Value
Integrated model	0.4418	0.7895	0.7568	0.8103	0.7179	0.8393

To evaluate the integrated model’s reliability and stability, we randomly divided the data into training and validation sets in a 7:3 ratio and implemented 100 iterations of resampling using the cross-validation method. The results showed that the integrated model’s accuracy, sensitivity, and specificity were satisfactory in both training and validation sets (**Table 6**).

**Table 6:** Integrated model verification results.

Group	Accuracy	Sensitivity	Specificity
Training	0.9272	0.9328	0.9183
Test	0.7719	0.8037	0.7250

## Discussion

M. C. Reyes et al. applied the concept of “implant morphology” from breast cancer images to patients with ovarian cancer. That is, in patients with BRCA mutations, the peritoneal implant morphology was dominated by “push” or circular contour, whereas in wild-type patients, invasive implantation was more common (15–18). In this study, on the basis of implant morphology combined with texture analysis, the primary tumor lesions were mapped in three dimensions to avoid the shortcomings of local sampling in genetic testing and ensure their diversity to the greatest extent possible. The quantitative texture features compensated for the deficiency of conventional CT-based empirical diagnosis.

Our multiple logistic regression analysis showed that nodular peritoneal metastases were significantly associated with BRCA mutation status, with a higher incidence in patients with the mutant type, whereas invasive peritoneal metastases and mesenteric involvement with supradiaphragmatic lymph node enlargement were more likely to occur in wild-type patients, as indicated by H. A. Vargas et al. (9). S. Nougaret et al. observed that the peritoneal site of metastasis was more likely in patients with mutant-type BRCA (14), but there was no significant correlation in that study, which may have been limited by its small sample size, and further verification is needed. We also found that the presence of peritoneal metastasis was the independent predictor with the best predictive efficacy, but its AUC value was only 74%, and its sensitivity was poor (62%), which may be related to the use of routine CT as the empirical diagnosis method. M. C. Reyes et al. observed that the characteristics of metastatic foci outside the ovary were related to the mutation status of the BRCA gene, and that the routine CT features of primary tumor lesions (e.g., margins, architecture) had no clear correlations with BRCA gene phenotype. This could have been partly because these genomic changes might have a greater impact on implant morphology outside the ovaries (9, 14, 15). We believe that if human direct access to information about primary tumor lesions is limited to the naked eye, judgments be made directly on the basis of the results of macroscopic observation, and any deviation may weaken our understanding of the disease. We attempted to implement 3D sketching of the primary tumor lesions, extract texture features, and identify mutation characteristics that have predictive value, but the prediction performance of those features was poorer. The AUC value of Venous_Waveet_HLL_Firstorder_Median with satisfactory intermediate and effective energy values was only 76%. Therefore, we conducted combined analysis of the routine CT features and texture features, meaning that the extra-ovarian metastases and primary tumor lesions were combined to establish a comprehensive prediction model. The combined model had significantly improved prediction efficiency: its AUC value reached 86%, which was significantly better than those of either routine CT features or texture features alone. This study had some limitations: (1) single-center retrospective study, external validation not present; (2) small sample size; (3) only evaluation of BRCA status, not patients’ HRD status.

To the best of our knowledge, this study is the first to combine the modeling of routine CT features with texture analysis. In proposing the present model, we seek more accurate information to facilitate prediction of gene status, which could make it possible to use noninvasive imaging evaluation as a complementary intervention to biopsy.

## Data Availability Statement

The raw data supporting the conclusions of this article will be made available by the authors, without undue reservation.

## Ethics Statement

The studies involving human participants were reviewed and approved by The First Affiliated Hospital of USTC. Written informed consent for participation was not required for this study in accordance with the national legislation and the institutional requirements.

## Author Contributions

WW and YZ contributed to conception and design of the study. MRL organized the database. YQG performed the statistical analysis. MRL and YQG wrote the first draft of the manuscript. MRL, WW, YZ, YQG, and MZL wrote sections of the manuscript. All authors contributed to the article and approved the submitted version

## Funding

This work was supported by the National Natural Science Youth Science Fund Project (No. 81501468), by the Fundamental Research Funds for the Central Universities (No. WK9110000002).

## Conflict of Interest

YQG is employed by General Electric Co. Ltd.

The remaining authors declare that the research was conducted in the absence of any commercial or financial relationships that could be construed as a potential conflict of interest.

## Publisher’s Note

All claims expressed in this article are solely those of the authors and do not necessarily represent those of their affiliated organizations, or those of the publisher, the editors and the reviewers. Any product that may be evaluated in this article, or claim that may be made by its manufacturer, is not guaranteed or endorsed by the publisher.
